# A comparative study of the socioeconomic factors associated with childhood sexual abuse in sub-Saharan Africa

**Published:** 2012-03-19

**Authors:** Ismail Yahaya, Joaquim Soares, Antonio Ponce De Leon, Gloria Macassa

**Affiliations:** 1 Department of Public Health Sciences, Mid-Sweden University, Sweden; 2 Department of Public Health & Biostatistics, University of Birmingham, UK; 3 Centre for Evidence-Based Global Health, Nigeria; 4 Division of Social Medicine, Department of Public Health Sciences, Karolinska Intitutet, Sweden; 5 Department of Occupational and Public Health Sciences, University of Gavle, Sweden

**Keywords:** Childhood Sexual abuse, sexual violence, sub-Saharan Africa, socio-demographic factors, demographic and health survey

## Abstract

**Background:**

Childhood sexual abuse (CSA) is a problem of considerable proportion in Africa where up to one-third of adolescent girls report their first sexual experience as being forced. The impact of child hood sexual abuse resonates in all areas of health. The aim of this study was to describe the prevalence of childhood sexual abuse and variations across socioeconomic status in six sub-Saharan countries.

**Methods:**

Datasets from Demographic and Health Surveys (DHS) in six sub-Saharan African countries conducted between 2003 and 2007 were used to access the relationship between CSA and socio economic status using multiple logistic regression models.

**Results:**

There was no association between CSA and education, wealth and area of settlement. However, there was contrasting association between CSA and working status of women.

**Conclusion:**

This study concurs with other western studies which indicate that CSA transcends across all socio economic group. It is therefore important that effective preventive strategies are developed and implemented that will cross across all socio-economic groups.

## Background

Sexual abuse of children occurs throughout the world and can occur in different circumstances and settings. It is a problem of considerable proportion in Africa where up to one-third of adolescent girls report their first sexual experience as being forced [1–3]. However, it has been largely neglected in research and very little data are available on childhood sexual abuse (CSA) in Africa. Sexual abuse of children is a silent epidemic as well as a public health issue with negative long term effects which vary across individuals. It also depends on the extent or the degree of the abuse [4]. It is recognised as a cause of severe distress among young people [5]. The embarrassment, shame or fear of being blamed and a desire to keep the abuse secret make disclosure uncommon. The most frequent explanations for the sexual abuse of children in sub-Saharan Africa (SSA) include rapid social change and the patriarchal nature of society [6]. The impact of CSA resonates in all areas of health: physical, psychological, including negative sexual outcomes [7–11]. Victims of sexual abuse in childhood are predisposed to recurrent or repeated abuse in childhood and adulthood [12–15]. Most of the existing studies in Africa are either limited to school settings or are country specifics [16–21]. School based studies are usually not nationally representative as high proportions of children still do not attend primary schools in African countries [22]. In addition, a significant proportion of children who are exposed to sexual violence in school are more likely to drop out [23]. Moreover, where studies have been done in specific settings, methodology and definition issues have limited comparability across settings [24]. There is the need for studies which are nationally representative, methodologically sound and comparable across countries in SSA. Therefore, the aim of this study was to describe the prevalence of childhood sexual abuse and variations across socioeconomic status in six sub-Saharan Countries.

## Methods

The data reported here were from the Demographic and Health Surveys (DHS) conducted between 2003 and 2008 in six countries (Ghana, Liberia, Nigeria, Uganda, Zambia and Zimbabwe) in SSA available as of November 2010. The countries were chosen based on the availability of data sets on childhood sexual violence (sexual violence). DHS surveys were implemented by respective national institutions and ICF Macro International Inc (Calverton, MD). Methods and data collection procedures have been published elsewhere [25]. The surveys were designed to collect good quality, nationally representative data on demographic and health indicators in most developing countries, with at least one woman of reproductive age (between 15 and 49 years) from each household. Sample sizes ranged between 5,000 and 15,000 households. Each domain is made up of enumeration areas (EAs) established by a National implementing agency in each of the countries. The sampling frame is a list of all EAs (clusters). Within each domain, a two stage sample was selected. The first stage involved selecting clusters (primary sampling units) with a probability proportional to the size, the size being the number of households in the cluster. The second stage involved the systematic sampling of households from the selected clusters. A standardised questionnaire was administered by interviewers to participants in each country. The survey's questionnaires were similar across countries making comparability across settings feasible.

### Outcome variable

For this study, CSA was defined as sexual violence on or before the age of 18 years. To assess if participants were sexually abused at childhood, all eligible women were asked the following questions: “At any time in your life, as a child or as an adult, has anyone forced you in any way to have sexual intercourse or perform any other sexual acts”? The two possible outcomes for the questions were “yes” or “no”. Respondents who said yes were then asked questions about the age at which this first happened and the person who committed the act. Respondents who responded yes and if the violence occurred when they were less than 18 years were considered as cases of CSA and coded as “1” while those who responded no or if it occurred after the age of 18 years formed the other group of the dichotomy and coded “0”.

### Determinants variables

**Socioeconomic factors:** This study considered four measures of socioeconomic positions: wealth index, occupation, education and place of residence. We selected these four socioeconomic factors based on previous studies that investigated factors associated with CSA and availability of the variables on the DHS. The wealth index was calculated using easy-to-collect data on household ownership of selected assets, such as televisions and cars; dwelling characteristics such as flooring materials; type of drinking water sources; toilet facilities; and other characteristics that are related to wealth status. They are then assigned a weight or factor score generated through principal component analysis. The weighted scores were divided into quintiles for the analytic models (lowest, middle, and highest). The level of education was assessed using a 3-point scale which reflected the highest level of education attained by the participants (no education, primary and secondary/ higher level of education). Women's current occupation was defined as not working or working. Place of residence was defined as rural or urban; age of respondents was categorized into 15-24, 25-34, and 35-49 years. The relationship of the respondents to the perpetrators of sexual abuse is considered to be partners, families, friends, strangers or others.

**Ethics:** The surveys were approved by the ICF Macro's Ethics Committee, USA and the Ethics Committee in each of the participating countries. All study participants gave informed consent before participation and confidentiality of all data was maintained.

### Statistical analysis

In the descriptive statistics, frequency tabulations were conducted to describe the distribution of correspondents. The key variables were expressed as percentages. This was followed by contingency table analysis to examine the impact of all potential predictors on CSA using chi-squared test. Univariate analysis (unadjusted) with pre-defined explanatory variables and CSA as the dependent variable were performed in the first instance. In the final model, multiple logistic regression analysis was used to examine factors associated with CSA, with all covariates entered simultaneously in the regression model. The results were presented in the form of odds ratio with significant levels and 95% confidence intervals.

Regression diagnostics were used to judge the goodness-of-fit of the model. They included the tolerance test for multi-collinearity, its reciprocal variance inflation factors (VIF) [26,27], presence of outliers and estimates of adjusted R square of the regression model. The largest VIF, lesser than 10, or a mean VIF lesser than 6, represented acceptable fit of the models [28]. Stata, release 11.1 for Windows (Stata Corp., College Station, TX, USA) was used for all analyses. All tests were two tailed and p-value less than 0.05 was considered significant.

## Results

### Sample characteristics

The study uses data from DHS conducted between 2005 and 2008 in six sub-Saharan Africa countries. Table 1 shows the countries, years of data collection, sample size and the reported childhood sexual abuse. The sample ranged from almost 5000 in Ghana to as more than 30,000 in Nigeria. In general, the study reported low levels of childhood sexual abuse. Reported cases of CSA were highest in Zambia (4.3%) and the least in Liberia (0.3%). The level of urbanisation varies across countries, ranging from 17% in Uganda to 44–45% in Zambia and Liberia. Table 2 illustrates the socio-demographic characteristics of the study participants. Most (38.0–45.8%) of the study participants were aged between 15–24 years. The level of education varies among respondents across the countries. Liberia recorded the highest respondents without education (41.8%) while Zimbabwe had the least number of non-educated (4.3%). Liberia recorded the least number of respondents unemployed (1.3%), while Zimbabwe recorded the highest number of respondents without a job (63%). Half of the respondents were distributed within the middle wealth status. Vast majority of the study participants lives in rural area. The perpetrators of the sexual abuse vary across the countries, with most of the perpetrators known to the victims.

**Table 1:** Description of data sets and selected demographic characteristics

	Year of survey	Sample size	Reported Childhood sexual abuse (%)
Ghana	2008	4916	0.8
Liberia	2007	7092	0.3
Nigeria	2008	33385	2.0
Uganda	2006	8531	0.4
Zambia	2007	7146	4.3
Zimbabwe	2005/06	8907	0.7

**Table 2:** Percentage distribution of selected characteristics

Variable	Ghana %	Liberia %	Nigeria %	Uganda %	Zambia %	Zimbabwe %
**Age**						
15–24	38.8	38.4	38.0	42.3	42.0	45.8
25–34	29.6	30.5	32.5	30.9	33.8	29.8
35+	31.7	31.1	29.5	26.8	24.2	24.5
**Education**						
No education	25.3	41.8	39.7	20.7	10.4	4.3
Primary	20.3	35.0	19.7	57.7	53.3	33.4
Secondary+	54.4	23.3	40.6	21.6	36.4	62.4
**Occupation**						
Not working	22.8	1.3	38.3	14.0	45.7	57.6
Working	77.2	98.7	61.7	86.0	54.4	42.4
**Wealth**						
Poorer	25.0	25.0	25.0	25.0	25.0	25.0
Middle	50.0	50.0	50.0	50.0	50.0	50.0
Richer	25.0	25.0	25.0	25.0	25.0	25.0
**Type of residence**						
Urban	44.0	45.0	31.4	17.0	44.5	36.0
Rural	56.0	55.0	68.6	83.0	55.5	64.0
**Perpetrator**						
Partners	63.5	42.5	28.0	63.3	32.3	75.4
Families	3.8	8.5	8.7	3.2	13.8	3.4
Friends	20.7	17.0	25.8	15.4	15.4	2.2
Strangers	7.7	6.9	25.1	13.6	28.7	3.1
Others	4.3	25.2	12.4	4.4	9.9	15.9

### Unadjusted results

Table 3 shows unadjusted association between CSA and socio-demographic factors. Compared to respondents aged 15–24 years, respondents aged 25–34 years or 35–39 years were less likely to have been a victim of CSA in all countries except in Ghana and Liberia. Level of education was not associated with CSA except in Nigeria and Zambia where those with higher level of education (primary, secondary and higher) were more significantly likely to have been victims of CSA. The relationship between CSA and occupation was mixed. Respondents working were at increased risk to have experienced CSA in Nigeria (odds ratio (OR) 1.24, 95% confidence interval (CI) 1.05 – 1.46) while respondents working in Uganda were at reduced risk (OR 0.40, 95% CI 0.19 – 0.84). The association was not significant in other four countries. Respondents in the highest wealth status were more likely to have experienced CSA in Ghana, Nigeria and Uganda. No statistically significant difference in the odds of CSA was found based on the type of residence in any of the countries. The offenders vary across countries and they were more likely to be families, friends or strangers.

**Table 3:** Multiple logistic regression results showing unadjusted and adjusted odds ratio for relationship between childhood sexual abuse and different determinant variables sub-Saharan Africa

	Ghana		Liberia		Nigeria		Uganda		Zambia		Zimbabwe	
	Unadjusted	Adjusted	Unadjusted	Adjusted	Unadjusted	Adjusted	Unadjusted	Adjusted	Unadjusted	Adjusted	Unadjusted	Adjusted
	OR (95% CI)	OR (95% CI)	OR (95% CI)	OR (95% CI)	OR (95% CI)	OR (95% CI)	OR (95% CI)	OR (95% CI)	OR (95% CI)	OR (95% CI)	OR (95% CI)	OR (95% CI)
**Age**												
15–24	1 (reference)	1 (reference)	1 (reference)	1 (reference)	1 (reference)	1 (reference)	1 (reference)	1 (reference)	1 (reference)	1 (reference)	1 (reference)	1 (reference)
25–34	0.85 (0.42–1.72)	0.9 (0.38–2.12)	1.26 (0.47–3.36)	0.56(0.14–2.18)	0.70 (0.59–0.84)	0.45(0.32–0.65)	0.31 (0.13–0.76)	0.28 (0.10–0.76)	0.82 (0.63–1.06)	0.62 (0.37–1.05)	0.38 (0.20–0.74)	0.18 (0.08–0.39)
35–49	0.43 (0.18–1.01)	0.84(0.3–2.36)	0.46 (0.12–1.75)	0.09(0.00–0.82)	0.44 (0.35–0.54)	0.38(0.25–0.59)	0.18 (0.55–0.60)	0.16 (0.04–0.61)	0.61 (0.44–0.83)	0.64 (0.34–1.21)	0.29 (0.13–0.66)	0.20 (0.08–0.51)
**Education**												
No education	1 (reference)	1 (reference)	1 (reference)	1 (reference)	1 (reference)	1 (reference)	1 (reference)	1 (reference)	1 (reference)	1 (reference)	1 (reference)	1 (reference)
Primary	1.66 (0.58–4.81)	0.7(0.2–2.52)	0.66 (0.22–1.98)	0.49(0.11–2.28)	3.19 (2.49–4.09)	1.09(0.66–0.54)	0.72 (0.36–1.44)	0.55 (0.20–1.52)	1.86 (1.13–3.04)	1.77 (0.78–4.00)	1.55 (0.94–2.55)	2.37 (1.19–4.70)
Secondary	2.02(0.83–4.93)	0.57(0.17–1.85)	1.00 (0.33–2.98)	0.66(0.11–3.81)	3.64 (2.93–4.52)	0.88(0.54–1.44)	–	–	1.96 (1.19–3.24)	2.00 (0.80–5.00)	–	–
**Occupation**												
Not working	1 (reference)	1 (reference)	1 (reference)	1 (reference)	1 (reference)	1 (reference)	1 (reference)	1 (reference)	1 (reference)	1 (reference)	1 (reference)	1 (reference)
Working	0.38(0.20–0.71)	0.34(0.15–0.77)	–	–	1.24 (1.05–1.46)	1.08(0.77–1.53)	0.40 (0.19–0.84)	0.24 (0.08–0.69)	0.93 (0.74–1.17)	0.45 (0.27–0.74)	0.80 (0.48–1.34)	0.67 (0.37–1.22)
**Wealth**												
Poorer	1 (reference)	1 (reference)	1 (reference)	1 (reference)	1 (reference)	1 (reference)	1 (reference)	1 (reference)	1 (reference)	1 (reference)	1 (reference)	1 (reference)
Middle	1.67(0.67–4.17)	1.14(0.37–3.49)	0.75 (0.27–2.11)	0.67(0.14–3.3)	1.19 (0.98–1.46)	0.66 (0.42–1.05)	2.25 (0.76–6.66)	0.84 (0.23–3.06)	1.24 (0.92–1.66)	1.09 (0.59–2.03)	1.03 (0.56–1.92)	0.98 (0.45–2.11)
Richer	2.35(0.90–6.13)	1.86(0.49–7.09)	0.67 (0.19–2.36)	0.48(0.04–5.99)	1.36 (1.09–1.69)	0.80 (0.44–1.43)	3.26 (1.06–10.01)	0.72 (0.15–3.35)	1.20 (0.86–1.68)	1.02 (0.40–2.62)	1.07 (0.53–2.16)	1.99 (0.56–7.06)
**Type of residence**												
Rural	1 (reference)	1 (reference)	1 (reference)	1 (reference)	1 (reference)	1 (reference)	1 (reference)	1 (reference)	1 (reference)	1 (reference)	1 (reference)	1 (reference)
Urban	1.41(0.76–2.63)	0.99(0.41–2.36)	0.89 (0.36–2.21)	1.28(0.23–7.12)	0.97 (0.82–1.14)	0.60(114–378)	1.22 (0.53–2.80)	0.59 (0.17–2.03)	1.08 (0.86–1.36	1.22 (0.65–2.28)	1.13 (0.67–1.88)	1.64 (0.67–4.02)
**Perpetrator**												
Partner	1 (reference)	1 (reference)	1 (reference)	1 (reference)	1 (reference)	1 (reference)	1 (reference)	1 (reference)	1 (reference)	1 (reference)	1 (reference)	1 (reference)
Families	23.98 (7.26–79.17)	16.7(4.7–59.39)	1.27 (0.25–6.34)	–	1.95 (1.09–3.51)	2.07(1.14–3.78)	5.2 (0.55–49.4)	3.83 (0.32–45.51)	4.78 (2.30–.95)	4.66 (2.17–10.00)	17.63 (7.84–39.65)	20.25 (7.94–51.63)
Friends	6.67 (2.73–16.32)	5.44(2.15–13.8)	1.27 (0.37–4.41)	1.96 (0.40–9.62)	1.38 (0.94–2.02)	1.4(0.94–2.07)	13 (4.02–42.09)	9.90 (2.91–33.69)	5.47 (2.64–11.29)	5.32 (2.51–11.31)	2.71 (0.60–12.28)	1.97 (0.40–9.72)
Strangers	8.88 (2.99–26.38)	9.86(3.05–31.84)	1.59 (0.31–8.02)	1.29 (0.12–13.78)	1.57 (1.07–2.32)	1.4(0.94–2.07)	15.05 (4.63–48.92)	10.13 (2.80–36.65)	3.40 (2.01–5.76)	3.52 (2.01–6.16)	18.31 (7.89–42.47)	27.86 (10.52–73.79)
Other	6.42 (1.55–26.55)	6.86(1.54–30.51)	0.41 (0.08–1.97)	0.51 (0.08–3.16)	2.01 (1.20–3.56)	2.22(13–3.79	44.57 (11.98–165.89)	40.73 (9.25–179.35)	8.16 (3.03–21.94)	7.83 (2.83–21.71)	1.43 (0.63–3.20)	1.60 (0.69–3.69)

OR: odds ratio, CI: confidence interval

### Adjusted results

Table 3 presents the results of adjusted odds ratio of factors associated with CSA. The diagnosis of multi-collinearity showed that the largest VIF ranged from 2.21 to 7.70; and the average VIF ranged from 1.46 to 2.37. Since none of the VIF values exceeds 10 and none of the average VIF exceeds 6, we concluded that there was no multi-collinearity problem. After adjusting for respondents socio-demographic factors, the association between age and CSA remained significant in Nigeria, Uganda and Zimbabwe. However, respondents aged 35–49 years were less likely to be victims of CSA compared to those aged 15–24 years. The association between education and CSA which was initially significant became non-significant in Nigeria and Zambia. Similarly, there was no statistical significant association between wealth and CSA in any of the six countries. Respondents in the working force were less likely to have been victims of CSA in Ghana (OR 0.34, CI 0.15 – 0.77), Uganda (OR=0.24, CI 0.08 – 0.69) and Zambia (OR=0.45, CI 0.27 – 0.74). It was only in Nigeria (OR 0.60, CI 0.42 – 0.87) that those living in the urban areas compared to the rural areas were less likely to have been victims of CSA. The perpetrators of sexual abuse vary across countries just as in the unadjusted analysis.

## Discussion

In this nationally representative cross sectional study of six countries in SSA, low levels of childhood sexual abuse was reported, with little associations with demographic characteristics. Findings presented in this paper show that older women were less likely to have experienced CSA. Further, our analysis found that currently working women were also less likely to have experienced sexual abuse in childhood.

The reported prevalence rates of CSA in this study fall below the lower range of reported rates typically reported in CSA studies, which reports between 10% and 25% of female [29–31] cases of CSA. There are possible explanations for this. Firstly, it might be related to the population characteristics and definitions used for CSA in the study. Child abuse operational definitions and the study response rates co-vary with response rates [32], i.e. a high response rate is associated with a relatively low prevalence rate. DHS are usually well conducted with a high response rate (average of 96%), and may as well contribute to the low prevalence of CSA in this study. In addition, the most restrictive definition of CSA was used; a definition that excluded non-contact sexual acts such as sexual request or exhibitionism. With this restrictive definition it is expected to have reduced prevalence of CSA. In addition, it might also be that the low prevalence was because of underreporting of cases, as study respondents may not be willing to disclose sexual abuse during the time of reporting [24]. The characteristic of perpetrators observed in this study is consistent with previous studies [6,30,33], that most of the perpetrators were known to the respondents prior to the episode of the abuse. Studies have shown that most abuse is committed by men (90%) and by persons known to the child (70–90%), with family members constituting one-third to one-half of the perpetrators against girls [34]. Most sexually abused children do not tell anyone they were abused, even when directly asked by parents or other authority figures. Victims of sexual abuse are often too afraid that the news will hurt their parents, or they are afraid of not being believed, or they were threatened in some way by the offender. The finding presented in our paper is consistent with the findings from other studies [30,35] that the risk of CSA is not related to socioeconomic factors. Rates of CSA were not significantly higher in families with low level of education, low wealth status and in rural communities. Nonetheless, there were instances where CSA was associated with higher level of education in Zimbabwe and small tendency for the risk of CSA to be related to type of residence in Nigeria.

There was contrasting association between CSA and occupation of respondents. Those reporting CSA less often were women currently working. This result is not consistent with previous findings [35] that have suggested that there was no linkage between CSA and socioeconomic factors. A possible explanation for this may be because the variable (occupation) was derived by combining all working groups together (official and non-official) versus those that were not employed. In such combinations, it is expected that each category of the groups could contain both high and low socio economic class.

This study is not without limitations. The DHS covered general health issues on population health with questions on sexual abuse constituting a minor part of the whole survey. With cross sectional study it is difficult to resolve the direction of causal association. The data were collected through self-report and it is likely that prevalence estimates obtained may be lower than the true prevalence because respondents may not disclose previous sexual abuse due to the sensitive nature of the questions being asked. Considering the accuracy in which respondents will be able to report CSA, it would be unrealistic that an exact account of the exposure to CSA will be available. The fact that the data used a cross-sectional design, limited the opportunity to deal with temporal ordering of abuse, risk behaviour and social marginality. Sexual abuse was limited to the time of onset and perpetrator of the abuse while precise information about the nature and extent of the abuse was not provided. Our study focused on socio-demographic characteristics as a predictor of CSA. We did not assess the effect of other predictors of CSA such as family stability and marital conflict, parenting and parent-child relationships and parental adjustment, as these variables were not obtainable from the DHS. There is a need for longitudinal studies that will identify all relevant variables and follow them up over a long period to identify relevant factors associated with CSA in SSA.

Despite the limitations, our study obtained strength in it being a large population-based study in six countries with a high response rate. It is a nationally representative sample with similar variables used across countries, making it possible for numerical values to be comparable across countries. More so, the data obtained from DHS are widely perceived to be of high quality based on sound sampling methodology and adherence to ethical standards of data collection including violence data.

## Conclusion

The findings from this study indicate that CSA is not linked to any socioeconomic background and that perpetrators of sexual abuse are known and close to the children abused. However, victims are usually not willing to disclose events and in the process leading to recurrent events. It is therefore important that effective preventive strategies are developed and implemented that will cut across all socioeconomic sphere in a context that both permit and encourages disclosure as well as identifying predisposing circumstances for recurrence.
